# Quantitative abilities of invertebrates: a methodological review

**DOI:** 10.1007/s10071-021-01529-w

**Published:** 2021-07-19

**Authors:** Elia Gatto, Olli J. Loukola, Christian Agrillo

**Affiliations:** 1 Department of General Psychology, University of Padova, Via Venezia 8, 35131 Padua, Italy; 2 Ecology and Genetics Research Unit, University of Oulu, POB 3000, 90014 Oulu, Finland; 3 Padova Neuroscience Center, University of Padova, Padua, Italy

**Keywords:** Animal cognition, Comparative psychology, Discrimination learning procedure, Invertebrates, Numerical cognition, Spontaneous choice test

## Abstract

Quantitative abilities are widely recognized to play important roles in several ecological contexts, such as foraging, mate choice, and social interaction. Indeed, such abilities are widespread among vertebrates, in particular mammals, birds, and fish. Recently, there has been an increasing number of studies on the quantitative abilities of invertebrates. In this review, we present the current knowledge in this field, especially focusing on the ecological relevance of the capacity to process quantitative information, the similarities with vertebrates, and the different methods adopted to investigate this cognitive skill. The literature argues, beyond methodological differences, a substantial similarity between the quantitative abilities of invertebrates and those of vertebrates, supporting the idea that similar ecological pressures may determine the emergence of similar cognitive systems even in distantly related species.

## Introduction

The concept of quantity is an abstract attribute that defines the fundamental attributes of the objects (Dehaene and Brannon 2011; Wiese 2003). This concept can be used in a wide variety of contexts, such as estimating the necessary time and distance required to travel, comparing the amount of food among available sources, or counting individuals in a social group. Historically, the use of quantitative information, especially numbers, was considered a unique human trait as a consequence of language (Dehaene 1992). However, growing evidence from developmental psychology and cognitive ethology has revealed that preverbal infants and nonhuman animals display the capacity to make quantitative estimations without symbolic representation (Feigenson et al. 2004; Nieder 2005). Taken together, these results suggest the presence of a common mechanism for the process of shared quantitative information among vertebrates that has presumably developed to solve several ecological contexts, such as foraging, mate choice, and social interaction (Shettleworth 2009).

Recent studies report the existence of quantitative abilities in invertebrates, resembling the ones shown by vertebrates (Giurfa 2019; Pahl et al. 2013; Skorupski et al. 2018). From an evolutionary point of view, these cognitive similarities raise important questions about the origin of quantitative abilities. Although humans and other vertebrates share a similar nervous system, invertebrates differ from vertebrates in this manner. Their last common ancestor lived at least 600 million years ago and presented a simplified nervous system adapted for faster responses from external stimuli, poor visual capacity, and a limited behavioral repertoire (Lowe et al. 2015). Despite all morphological and functional neural differences, it has been suggested that vertebrates and invertebrates have developed similar cognitive functions without a last common ancestor possessing it (Chittka and Niven 2009; Chittka et al. 2012). In the case of quantitative abilities, it is possible that similar ecological pressures acted in the development of quantitative abilities of vertebrates and invertebrates.

**Fig. 1:**
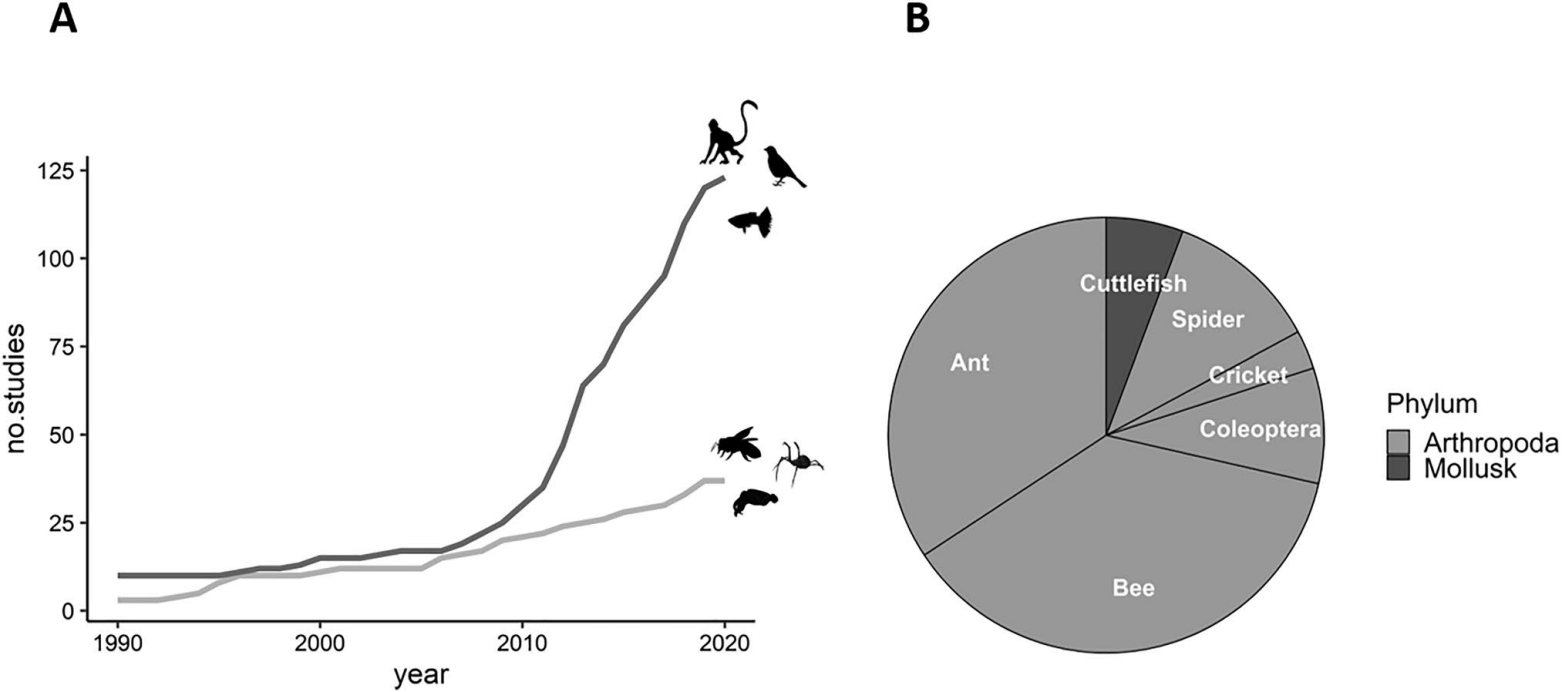
**A** Cumulative number of publications on quantitative discrimination abilities in vertebrates and invertebrates. The graph shows the cumulative number of publications returned from a search for (‘numerical abilities’ + animals) or (‘numerical competence’ + animals) on PubMed. The search was conducted on 12 March 2020. **B** Percentage of species investigated for the quantitative ability of invertebrates. Despite two studies on mollusk (dark grey), the majority of studies in the quantitative ability of invertebrates have mainly investigated in arthropods (light grey)

Recently, there has been growing interest in the quantitative abilities of invertebrates (Fig. 1A). Several reviews have been conducted on the topic (e.g., Giurfa 2019; Skorupski et al. 2018) and about the potential neural circuit underlying such capacity (Giurfa 2019; Nieder 2019, 2021). Researchers in the field have mainly focused on a few studied species, such as bees and spiders (Fig. 1B). Moreover, specific methodologies to investigate quantitative abilities in invertebrates have commonly been tailored to the study’s aim. Thus, the performance might be due to an innate predisposition of one species to solve a specific task (Shettleworth 1972), rather than a common cognitive function among species. Different and specific methodologies raised problems of validity in generalizing exceptional cognitive performance between and within the same species (Agrillo and Miletto Petrazzini 2012). To provide possible solutions for these issues, we reviewed the methods that have been used in studying the quantitative information use of invertebrates and highlighted the potential problems in the field. In the first section, we briefly provided a definition of quantitative ability, focusing on the current debate of the existence of one or two number systems. In the second section, we explained the ecological pressures that have led to the development of quantity discrimination abilities in invertebrates. In the third section, we reviewed the methodological approaches used to study quantitative abilities in invertebrates. In the fourth section, we discussed the advantages of studying quantitative abilities in invertebrates and the potential direction of further studies.

## A common number system?

Quantitative discrimination ability has been broadly investigated in several vertebrates and, more recently, in invertebrates. Thus, a single definition of ‘number’ could be limited and incomplete (Rezikova and Ryabko 2011). However, the mechanism underlying such capacity is still under debate and deserves more attention. Indeed, conflicting results do not support the hypothesis of one system or two systems based on numerical competencies (Henik 2016).

The first hypothesis concerns the existence of a single system, defined as the approximate number system (ANS), which is involved in processing small (e.g., range 1–4 items) and large numerosity ranges (e.g., > 4 items Gallistel and Gelman 2000; Brannon and Roitman 2003; Cantlon et al. 2009). The imprecision of ANS does not depend on an absolute limit of processing elements, but it is related to the numerical ratio between sets, defined as the ‘numerical distance effect’ (Gallistel and Gelman 1992). This confounding effect is related to the ‘distance effect’ [i.e., it is easier to discriminate between numerosity when they are numerically dissimilar (5 vs. 10 items) than when they are similar (5 vs. 6 items)], and the ‘size effect’ [i.e., the comparison between pairs of small numbers (2 vs. 3 items) is easier than pairs of large numbers (11 vs. 12 items)]. According to this hypothesis, the ANS estimates small and large numerosity ranges in a similar way because number magnitudes are represented as a point positioned along a continuous number line (e.g., Dehaene 2003). Thus, the estimation capacity depends on the numerical ratio rather than the absolute difference (Beran 2007; Dehaene and Brannon 2011; Gallistel 1989; Halberda and Odic 2015).

Inter- and intraspecies differences in estimating quantities between the small and larger number ranges have led some researchers to hypothesize the existence of a second system devoted to processing small numbers (reviewed in Agrillo et al. 2015a). The second system, defined as the object–tracking system (OTS), is based on a mechanism that permits the identification of every single object as a unique element different from the others (Trick and Pylyshyn 1993). The idea of this system was developed from visual attention theories in humans: the FINST model (Pylyshyn and Storm 1988) and the object–file model (Kahneman et al. 1992). The principal component of OTS is a primitive attentive mechanism that permits encoding a limited number of objects as separated elements stored in the working memory (Trick and Pylyshyn 1993). Thus, the second system operates differently than the ANS because it is involved only in estimating the range of small numbers due to the limit of working memory. In contrast to the ANS, OTS accuracy does not depend on the numerical ratio; rather, it depends on the quantity of representation stored in the working memory, which is proposed to be approximately three or four elements (Burr et al. 2010; Hyde 2011; Pylyshyn 2001; Agrillo et al. 2015b). Comparative studies have reported high performance on small number of discrimination tasks in humans (Feigenson et al. 2004), primates (Beran and Parrish 2016), birds (Garland et al. 2012), salamanders (Krusche et al. 2010), and fish (Agrillo et al. 2012). These studies support the existence of two systems involved in quantitative estimation (Feigenson et al. 2004; vanMarle 2015; Xu 2003).

The contrasting results in discriminating small and large numbers may be explained by the nature of stimuli. VanMarle (2015) has recently proposed that the ANS is used to estimate small number ranges when the physical properties of stimuli are modified (i.e., sensory modality). Thus, an individual is required to extract the concepts of the number to a solve task. The amodal stimulus may not activate the OTS because its mechanism involves visual and attentive perception of the stimuli’s physical attributes. Hence, the activation of the ANS for estimating small numbers relates to the experimental condition rather than the existence of a single system. Despite the large variation of numerical competence in vertebrates, many researchers are inclined to accept the hypothesis of a single magnitude system for processing quantitative information (e.g., Beran et al. 2015; Cantlon and Brannon 2007; Rugani et al. 2013).

## The ecological relevance of the quantitative ability of invertebrates

Animals face many situations in which being able to discriminate between quantities could be especially adaptive to maximize their fitness in relation to the ecological context. The access to limited resources may be costly in respect to energy expenditure, time loss, and susceptibility to parasites and predators; thus, selection should favor accurate information gathering to enable appropriate tactical decisions (Arnott and Elwwod 2009). The benefit is most obvious when animals display foraging behavior; the ability to identify the number of food sources present is relevant for the decision-making process. For instance, animals may maximize the quantity of food in relation to the cost of finding a food source by selecting the larger amount of food (Hauser et al. 2000; Lucon-Xiccato et al. 2015) or the option associated with minimal costs (Pantaleeva et al. 2013). Bumblebees (*Bombus terrestris*) increase their foraging efficiency by making decisions based on previously visited flowers by exploiting numerical regularities in the distribution of the flowers (Bar-Shai et al. 2011). Spider-eating spiders (*Portia africana*) evaluate the potential cost associated with the abundance of conspecific competitors when settling at the prey nest (Nelson and Jackson 2012). The redwood ants (*Formica* sp.) are characterized by a complex social system in which team communication is essential in solving nest necessities. After returning to the nest, scout ants communicate the food location to the foragers. The duration of time spent by the scouting ants for transferring information regarding food is related to the distance between the nest and sources (Reznikova and Ryabko 1993, 1994).

The assessment of rivals might be useful for social species in defending their territories because responses against rival groups (i.e., fight or escape) depend on the numerosity and size of companions and rivals (Shettleworth 2009). Two competing ant species, *F. xerophila* and *F. integroides*, modulated their fighting behavior by assessing the number of competitors (Tanner 2006). When subjects perceived themselves as a part of the larger group, they were more aggressive toward competitors than those perceived to be in the minority or isolated. Females of the two-spot ladybird (*Adalia bipunctata*) maximized their fitness by assessing the number of conspecific competitors. This species laid eggs near an aphid population, which guaranteed food to their offspring. Females assessed whether an aphid population was suitable for ovipositing based on the number of other gravid females that could limit the food resources for their own larvae. In the presence of competitors, individuals laid fewer eggs or delayed the oviposition for further favorable conditions (Hemptinne et al. 1992).

To reach food and other resources, animals need to travel between their safe refuges and potential risk places without losing their position within their environment. An efficient navigational system provides benefits in terms of rapid and safe travel, a reduction of energy, and time reaching the goal. Navigation requires a sophisticated capacity for spatial orientation, permitting an individual to assess their direction and position continuously with respect to their starting point and the destination (Heinze et al. 2018). The mechanism underlying this capacity is defined as ‘path integration’. During navigation, individuals must continuously keep track of their relative positions in the travel path by detecting cues emanating from their goals (e.g., floral scent), acquiring and using environmental information (e.g., polarized light), or learning sequences of behavior (Heinze et al. 2018). In the desert ant *Cataglyphis *sp*.*, individuals navigate by combining information from the path integration mechanism and memorizing panoramic visual cues. In this ant genus and other Hymenoptera (e.g., ants, bees, and wasps), the path integration mechanism detects the direction through a skylight compass using the pattern of polarized light, which infers the sun’s position and other celestial cues and measures the distance through a counting mechanism (reviewed in Freas and Schultheiss 2018; Heinze et al. 2018; Rössler 2019). The counting mechanism, defined as ‘odometer,’ estimates the distance based on the memorization of previous travel and the locomotion type. In walking ants, such as *Cataglyphis *sp*.*, the accumulated odometer information during foraging trips relies on the number of steps (Wittlinger et al. 2006, 2007). Foraging bees and other flying insects estimate the distance information from monitoring the optic flow, which is the pattern of objects’ apparent motion in a visual scene across their retinas when flying (Collet and Collet 2000; Heinze et al. 2018).

In a sexual choice context, males typically compete for access to females while females are choosy in selecting among males that may provide more benefits for their offspring (Andersson 1994). Whether a male is chosen might depend on a combination of factors in addition to his behavior and morphology, such as the availability of nest sites, food, or other resources in his territory. In many birds, male mating success is related to aspects of his territory size or quality. For example, male red-winged blackbirds (*Agelaius phoeniceus*) providing extra food in their territories attract more females (Ewald and Rohwer 1982; Wimberger 1988). In *Anthidium* bees, the male defends flowers as food sources and permits a female to feed only if she mates with him. Males attract females in proportion to the number of flowers defended (Alcock et al. 1977; Severinghaus et al. 1981). Male individuals of *Tenebrio molitor* maximize their fitness by selecting the female group with the best sex ratio (Carazo et al. 2009). *T. molitor* males perform individual recognition based on olfactory cues. When finding a female, the time spent on mate-guarding correlates positively with the number of other male competitors (Carazo et al. 2012).

Thus, it seems clear that the ability to discriminate between quantities positively contributes to the survival and reproduction of individuals. Given the importance of such cognitive abilities, invertebrates may develop a mechanism of quantity estimation to solve ecological challenges similar to those with which vertebrates deal (Table 1). Such cognitive similarity has raised the interest of researchers to investigate quantitative abilities in invertebrates systematically.

**Table 1:** Summary of the ecological contexts in which quantitative discrimination abilities have been studied in invertebrates

Contexts	Species	References
Abstract stimuli	*Apis mellifera* (honey bees)*, Bombus terrestris* (bumblebee), *Formica polyctena* (ant)	Leppik (1953), Gross et al. (2009), Skorupski et al. (2018), Howard et al. (2018, 2019a, b, 2020a), Bortot et al. (2019), Maboudi et al. (2020) and Reznikova and Ryabko (1993, 1994, 1996, 2000, 2001)
Foraging	*Apis mellifera* (honey bees)*, Bombus terrestris* (bumblebee)*, Nephila clavipes* (spider)*, Portia africana* (spider)*, Sepia pharaonic* (cuttlefish)	Bar-Shai et al. (2011), Nelson and Jackson (2012), Rodríguez et al. (2015), Yang and Chiao (2016) and Huang et al. (2019)
Navigation	*Apis mellifera* (honey bees)*, Bombus terrestris* (bumblebee)*, Cataglyphis fortis* (ant)	Chittka and Geiger (1995a; b), Chittka et al. (1995), Wittlinger et al. (2006, 2007), Dacke and Srinivasan (2008) and Menzel et al. (2010)
Nesting decision	*Leptothorax albipennis* (ant); *Myrmecina nipponica* (ant)*, Temnothorax albipennis* (ant)	Pratt et al. (2002), Franks et al. (2006) and Cronin (2014)
Predator avoidance/search for refuge	*Acheta domesticus* (cricket) *Theba pisana* (snail)	Gatto and Carlesso (2019) and Bisazza and Gatto (2021)
Social interaction	*Adalia bipunctata* (ladybird)*, Formica xerophila* (ant)*, Pardosa ramulosa* (spider), *Tenebrio molitor* (cockroach)	Hemptinne et al. (1992), Tanner (2006); Greenstone (1978) and Carazo et al. (2009, 2012)

## Methodologies for studying quantitative abilities in invertebrates

Although the number of studies that directly investigated quantitative abilities in invertebrates is limited, the experimental procedures are based on those used for investigating such capacities in vertebrates.

### Spontaneous choice test

The spontaneous choice test implies an unconditioned discrimination between biologically relevant stimuli. Animals are generally tested in their natural environments or in the laboratory under seminatural conditions. This procedure allows for studying the inherent quantitative abilities of a species in a situation that mimics its natural habitat without reinforcement or prior training. Observing how an animal behaves freely in critical conditions makes it possible to advance hypotheses on the adaptive importance of quantitative abilities. The capacity of making adaptive quantity discrimination in invertebrates has been investigated by exploiting their spontaneous preferences for a wide range of different stimuli.

#### Food choice paradigm

Food sources are probably the most used stimuli to investigate the spontaneous quantitative abilities of animals easily. Individuals are generally faced with a dichotomous choice between two different food quantities. Rodríguez et al. (2015) reported that golden orb-web spiders (*Nephila clavipes*) regulated their prey-searching behavior proportionally to the number of prey they lost, suggesting that this species can track and memorize the total amount of hunted prey accumulated in their webs. During foraging visits, bees can spontaneously discriminate among patches of flowers to maximize their foraging efficiency. Howard et al. (2020a) tested forager bees for their spontaneous preference by presenting 13 different quantity discriminations. Stimuli were artificial flowers (i.e., yellow circle) that differed for the number of items and overall surface area. Authors recorded the first 10 choices to evaluate spontaneous quantitative abilities. Results showed that bees spontaneously preferred the larger quantity only when a one-flower stimulus was presented as the smaller quantity in a relative quantitative task.

A preference for a specific quantity of food is not the same as expressing a preference for more (or for less). Optimal foraging theory may encourage thinking that choosing more is fundamental, but there are also circumstances where choosing fewer is more advantageous. In two studies, the quantitative ability of cuttlefish (*Sepia pharaonic*) has been investigated in a spontaneous choice test by exploiting their predatory behavior. Subjects were presented with a dichotomous choice between groups of shrimp that differed by numerosity or prey quality (e.g., size of prey and live versus dead prey). Cuttlefish preferred the largest number of prey, but they chose the small group when the choice was between one live or two dead shrimp. They also chose a single large shrimp over two smaller ones (Huang et al. 2019; Yang and Chiao 2016). This evidence has interesting implications because cuttlefish simultaneously use different types of information when making foraging decisions, such as the size or quality of the food source.

#### Social context

Foraging is not the only domain in which animals show quantitative abilities. For example, social stimuli instead of food items are used in a context where it is necessary to estimate the number of potential rivals. Individuals of *Formica xerophila* can compare the relative quantitative difference between their group and a competing group. Subjects that perceived themselves as a part of the largest group are more aggressive toward competitors than are those that perceive themselves as isolated (Tanner 2006). Reznikova and Ryabko (1993, 1994, 1996, 2000, 2001, 2009) developed an experimental paradigm for studying quantitative abilities in red wood ants by exploiting their communication skills when foraging. Like several other ant species, redwood ants are characterized by a highly social system in which different groups are predisposed to solve specific tasks for the colony. Scout ants explore the environment and search for food. When finding any food source, scout ants communicate the food location to the foragers. Groups of foragers reach the sources and collect the food. The researchers investigated the duration of communication by comparing the colonies in which scouts were trained to find food sources at different distances. Findings showed that scout ants spent more time communicating information to the foragers when the travelled distance was longer.

#### Mate choice

Furthermore, estimating the number of mate rivals can maximize an individual’s fitness. Carazo et al. (2009) studied male mate choice in mealworm beetles (*T. molitor*) using a spontaneous choice test in which males were exposed to two substrates bearing odors from different numbers of female conspecifics. Males exhibited a preference for the sources reflecting numerous different females. In the following experiment, the authors found that male *T. molitor* adjusted their investments in mate guarding according to the number of different rival males (Carazo et al. 2012). These experiments suggest that visual cues are not necessary for making quantitative decisions. *T. molitor* is a polygynandrous plant scavenger that uses chemical signals for individual recognition. In particularly, males have several strategies in response to high sperm competition (e.g., Carazo et al. 2004; Drnevich 2003; Drnevich et al. 2000; Griffith 2001; Happ 1969). Assessing male abundance by individual recognition seems advantageous to evaluate the risks and levels of competition. Indeed, males adjust the amount of time they allocate to mate guarding according to the abundance of competitors. Individual recognition is mediated by the abundance and variety of chemical cues (Carazo et al. 2004). Even though these studies are not definitive, they challenge the hypothesis of a cross-modal system for quantity discrimination.

In several species of ladybird, gravid females lay eggs directly in aphid colonies to provide immediate food sources to support the development of their offspring. Females of the two-spot ladybird (*Adalia bipunctata*) inhibit egg laying in the presence of a high number of conspecific gravid females as rival competitors (Hemptinne et al. 1992). Nelson and Jackson (2012) investigated quantitative abilities on predation strategies of spider-eating spiders (*P. africana*). This spider species performs communal predation when searching for prey, generally with another spider species, *Oecobius amboseli*. Individuals settle and wait near already present conspecifics. Nelson and Jackson (2012) found that *P. africana* based their settling decisions according to the number of conspecifics and minimized the competition by settling when the number of conspecifics was one instead of zero, two, or three.

#### Spatial navigation

The ability to learn the location of places in the environment is an essential aspect of individual life. When foraging, individuals learned source positions based on several types of information (i.e., visual, olfactory, physical, and magnetic cues) to minimize the cost of navigation. Wittlinger et al. (2006, 2007) found that ants of the *Cataglyphis fortis* species measured distance with a “step counter” system during a navigation task. Ants were trained to reach a feeder at a fixed position from the colony. Authors manipulated the extension of the ant foragers’ legs by shortening or lengthening the tarsi. Test subjects traveled shorter or longer lengths than control subjects did, suggesting this species use a system based on integrating step counts during navigation (Wittlinger et al. 2006, 2007). The presence of an innate odometer system also has been reported in the wolf spider *Lycosa tarantula*. Such species use path integration based on angular and linear displacement. Ortega-Escobar and Ruiz (2014) investigated how *L. tarantula* estimates the linear component of path integration. In a series of consecutive trials, subjects were moved the same distance into a tunnel to reach their home. In the test trial, the authors manipulated subjects’ perceptions of the visual stimuli presented in the tunnel (i.e., the optic low supplied by a pattern of black and white stripes). Results showed that *L. tarantula* used visual information for quantifying encompassed distance.

In dangerous contexts, such as in predator avoidance contexts, animals make antipredator decisions by estimating the costs and benefits of escaping and hiding. The house cricket *Acheta domesticus* is a small nocturnal cricket that actively searches for burrows in an open field as potential refuges in which to escape from predators (Kieruzel 1976). Gatto and Carlesso (2019) exploited the natural shelter-seeking behavior of *A. domesticus* by presenting a set of stimuli that mimicked potential refuges. Similar to a procedure adopted for study quantity discrimination in treefrogs (Lucon-Xiccato et al. 2018), this approach permits carefully manipulating specific variables of stimuli to investigate which attributes are relevant for the individual when estimating quantities. Authors found that house crickets selected sets containing a larger numerosity up to two versus three bars, and crickets attended more to the width than the height of the stimuli when making quantity discrimination. *Theba pisana* is a small snail that inhabits a harsh environment, the coastal dunes of the Mediterranean Sea, characterized by sparse vegetation, sandy soil, and elevated diurnal temperatures. To avoid dehydration because of the elevated temperatures, *T. pisana* must locate and reach tall vegetation as an elevated refuge position from the soil. Natural selection has driven the evolution of a system that permits this snail species to find a suitable group of vegetation rapidly to escape from the soil temperatures. Based on their ecology, Bisazza and Gatto (2021) investigated whether *T. pisana* would prefer larger rather than smaller groups of vertical bars in a fully bright condition, a situation that simulated the natural habitat of this species. In subsequent laboratory experiments, authors found that the numerical acuity of snails reached the four- versus five-item discrimination, a numerical performance comparable to that exhibited by many vertebrates.

#### Expectancy-violation paradigm

Finally, the spontaneous quantitative ability of invertebrates has been studied by adopting methods based on the expectancy-violation theory. Such methodologies have been especially prominent in research on the numerical capacities of human infants, nonhuman primates, and parrots (Wynn 1992; Hauser et al. 1996; Pepperberg and Kozak 1986). In these cases, subjects have been shown addition and subtraction operations. For example, an item or a collection of items has disappeared behind an obstacle, or items have been moved from one set to another. During these experiments, the expected outcome has been consistent with the observed operations in some cases but inconsistent in others. Individuals usually take longer times on unexpected events; usually, this has been considered a measure of the spontaneous ability to make calculations because subjects detected a mismatch between the current scenario and the expected one (Wynn 1992). Such an innate capacity does not necessarily require an understanding of the abstract concept of numbers or the training procedure to perform quantitative discrimination (Nieder 2020).

The expectancy–violation paradigm was successfully adopted in the research on *P. africana* (reviewed in Cross et al. 2020). Cross and Jackson (2014) adopted an expectancy-violation method for investigating whether the jumping spider *P. africana* can represent a specific prey type during predatory sequences. At the beginning of the trial, subjects were presented with a particular prey species (i.e., *Arachnura scorpionoides*, *Argyrodes *sp*.*, and *Pycnacantha tribulis*) positioned in different orientations. Before subjects could attack, the prey was hidden behind an obstacle. After a 90-s delay, the obstacle was removed, and the subject was able to see the prey. The experimenters compared the length of time before subjects attempted to attack the prey in consistent (no changes) and inconsistent (prey change) events. Changing the orientation of prey did not affect the subjects’ predisposition to attack. However, when the prey species or prey color changed, many *Portia* individuals looked longer at the prey before approaching it. Based on their results, Cross and Jackson (2014) suggested that *P. africana* represented prey type by subitizing, this being the comparison of the representation of the prey stored into working memory at the beginning of a trial and the unexpected type.

In a second study, Cross and Jackson (2017) adopted a different variant of their previous study (2014) for investigating whether *P. africana* represented exact numbers of prey. The experiments concerned the spider’s capacity to gain access to prey by exploring a preplanned detour maze in which the prey’s sight was not always accessible to the subjects. At the beginning of the maze, subjects first viewed a scene with a particular number of prey positioned at the end of the maze. Then, they took a series of detours to reach the prey. When the subjects reached the scene, the number of prey items might have been different. According to the expectancy–violation hypothesis, *P. africana* showed different behavior when facing an unexpected scene, suggesting the capacity to make spontaneous operations without any training.

### Discrimination learning

Discrimination learning procedures are based on the concept of operant conditioning introduced by Thorndike at the beginning of the twentieth century (Hall 2002). Operant (or instrumental) conditioning is a methodological procedure in which individuals learn the association between the behavior (i.e., response) and outcome (i.e., punishment or reward). Learning, especially associative learning, is an adaptive mechanism enabling individuals to solve unexpected events in their environments through previous experiences (Maren et al. 2013).

In discrimination learning procedures, animals are initially trained to respond to each of the two or more neutral stimuli. Neutral stimuli are commonly associated with a food reward or punishment. Then, subjects are tested in a novel discrimination task by applying the learning rule. This experimental approach required sophisticated apparatus and prolonged training, but it allowed for the administration of an elevated number of trials per subject in standard conditions (Agrillo and Bisazza 2014). Studies on mammals and birds included thousands of reinforced trials with remarkable performances similar to one exhibit by humans (Beran 2008; Emmerton and Delius 1993; Tomonaga 2008). Second, the standardized conditions permit comparing performance to study similarity and difference of the mechanisms underline quantitative abilities among species (Cantlon and Brannon 2006, 2007). Third, the use of artificial stimuli, generally projected from a monitor, permits a detailed manipulation of the features of stimuli to control the confounded effects of continuous variables (Agrillo and Bisazza 2014).

Most of the training procedures involved the foraging activity of invertebrates during navigation. Individuals are generally trained to forage from a feeder in an open field or in a maze at a fixed distance. To find the feeder, individuals need to extrapolate quantitative information provided by landmarks. Several studies report the ability of bees to find the reinforced food source using the number of landmarks passed during the flight (Chittka and Geiger 1995a, b; Chittka et al. 1995; Dacke and Srinivasan 2008; Menzel et al. 2010). The capacity of abstracting or relying on contextual cues has been widely investigated in bees by adopting a delayed-matching-to-sample protocol (DMTS). In this procedure, bees are generally trained to fly into a Y-maze and memorize a spatial array positioned at the maze entrance (i.e., reference stimulus). Bees are faced with a dichotomous choice between spatial arrays with different numbers of items. To find a food source, bees have to choose the array that contains the same number of elements as the reference. To solve this task, bees need to understand the abstract concept of classification. Stimulus classification can be achieved by considering common physical features of the reinforced category of stimuli (Zentall et al. 2002). However, the concept of “better than” or “worse than” required individuals to learn beyond the perceptual generalization of stimuli because subjects needed to understand the abstract concept at the base of the relation with two or multiply stimuli, defined as relational classification or relation learning (Zentall et al. 2002). In the DMTS protocol, subjects need to acquire the physical differences between stimuli (such as color, shape, or number of elements) and then categorize a new stimulus according to the relation between the learned categories. Gross et al. (2009) trained bees in the DMTS paradigm to make decisions about the sameness or difference of visual arrays based on the quantitative information (i.e., number of objects present in each stimulus). Trained bees successfully discriminated arrays with two or three elements, and array variations in color, shape, or configuration had no impact on the bees’ performances, suggesting that bees made decisions based on quantitative information. Recent studies have provided further evidence on the quantitative abilities of bees using a similar approach to Gross et al. (2009). Howard et al. (2018, 2019a, b) trained bees to choose quantities according to the rules of “greater than” and “less than”, showing that bees can make quantitative discrimination and even understand the concept of zero as an element on a number line. Bees were initially trained to understand the concept of “numerical less”, a dichotomous choice between white squared-cardboard stimuli containing 1–4 black items. Then, they required extending the numerical rule to a novel set of stimuli. Bees were able to extend these concepts to discriminate between sets of elements in the small number range, and bees even demonstrated the representation of an empty set (i.e., white cardboard without elements) as the lower element of a numerical continuum.

In a recent experiment, MaBouDi et al. (2020) trained bumblebees with a different conditioning approach. The novelty of this approach was related to the presentation of multiple couples of spatial arrays differing with respect to the number of items they contained. This situation resembles the natural context in which bees forage among multiple food sources. These authors trained bees to choose sets of spatial arrays containing two or four items. With this procedure, bees learned the task independently of the color or shape of the elements or the areas subtended by them. Analyses of the bees’ flight paths show interesting evidence about decision-making processes. Bees scanned all items within an array one by one before making a decision, suggesting that bees memorize and process integrating information similar to the working memory system possessed by humans and nonhuman primates.

## Future directions

### Different methodological approaches may underlie different performances in quantitative tasks

In examining the evidence reported in this review, one may be tempted to conclude that invertebrates have a rudimentary system of quantitative representation similar to one found in vertebrates. However, before accepting this idea, it is necessary to note that quantitative abilities have been studied in invertebrates by adopting quite different methods. Hymenopterans, especially bees, have been trained with prolonged procedures adopted from methods used to test the numerical abilities in vertebrates (Howard et al. 2018, 2019a, b; Maboudi et al. 2020). However, the spontaneous choice test is the most common procedure used in studies to focus on the quantitative abilities in other invertebrates, such as cockroaches, cuttlefish, and spiders (Carazo et al. 2009; Nelson and Jackson 2012; Yang and Chiao 2016). The variety of specific procedures and limited number of studies in some species may affect the assessment of such capacity. Recent studies in fish reported that different methods can affect the measuring of quantitative abilities (Agrillo and Dadda 2007; Gatto et al. 2017; Lucon-Xiccato et al. 2017), as is also the case for other cognitive abilities (Prétôt et al. 2016; Salwiczek et al. 2012). Comparative behavioral studies provide a useful tool for investigating cognitive differences and similarities among species, but it is necessary to be careful about the methods that are adopted to assess such capacities. Interspecific comparison requires standardizing the methods used with different species (Agrillo and Bisazza 2014). Moreover, methodological differences within invertebrates might arise due to difficulties in developing a procedure to study complex cognitive abilities in other invertebrate taxa than hymenopterans. For example, bumblebees (*B. terrestris*), which are constantly motivated on foraging, can be trained to solve complex tasks to obtain a food reward (Alem et al. 2016; Loukola et al. 2017). However, these kinds of training procedures based on foraging activities may not be extended to all invertebrates, especially if the species are motivated on foraging only occasionally. As suggested by Gallistel (1989), human and nonhuman animals possess a common system that is activated whether it is necessary to discriminate between quantity in various domains (i.e., number, space, and time). To test this hypothesis, we need to extend the number of investigated species, especially invertebrates, and the contexts in which quantitative information may be useful.

### Discrete versus continuous variables in numerical discrimination

Studies focusing on quantitative abilities on invertebrates have not gone especially far toward disentangling which attributes are exploited in quantity discrimination, even though some authors have conducted carefully controlled experiments for ruling out the role of continuous variables (Bisazza and Gatto 2021; Cross and Jackson 2017; Gatto and Carlesso 2019; Howard et al 2018; MaBouDi et al. 2020; Nelson and Jackson 2012; Yang and Chiao 2016). Indeed, discriminations based on discrete variables (e.g., identifying each single item within a group) or on analogue variables (e.g., continuous features of stimuli as the overall surface encompassed by stimuli) show comparable results, but it is necessary to investigate carefully which attributes are attended to before claiming that a species has the capacity for processing numerical information (Feigenson 2007).

Neutral or nonbiologically relevant stimuli, such as two-dimensional geometrical figures or dots projected from a monitor, can be manipulated for most of the continuous variables and have been used in investigating whether animals rely on discrete variables when making quantitative decisions. Franks et al. (2006) used a spontaneous choice test for investigating whether an ant species (*Temnothorax albipennis*) discriminated between nests differing in the number of available entrances. Results suggested that this ant species could discriminate between different numerosity. However, individuals could have relied on different physical features of entrances, such as light intensity or entrance size. Control experiments revealed that individuals of this ant species mainly relied on the summation of light levels within nesting entrances rather than the number of entrances.

Gatto and Carlesso (2019) investigated spontaneous quantitative discrimination of house crickets (*A. domesticus*) using two-dimensional geometrical shapes that resemble potential refuges. By exploiting the natural shelter-seeking behavior in dangerous situations, the authors found that crickets showed a preference for larger groups of up to two versus three elements. Crickets were tested with two-dimensional stimuli using methods that facilitated controlling for the potential confounding influence of continuous variables. The aim of this study was to study whether crickets could discriminate between quantities and which quantitative information was more relevant. Crickets were presented with a dichotomous choice between sets of geometrical figures that resembled potential refuges. Indeed, the experimental set-up elicited the escape responses of crickets. The authors manipulated the discrete and the continuous variables of stimuli by presenting stimuli that differed by numerosity and/or size. Crickets showed a preference for the larger stimulus by focusing mainly on continuous quantities rather than numerical information.

Similarly, Bisazza and Gatto (2021) investigated quantitative discrimination abilities in terrestrial snails (*T. pisana*) by presenting groups of stimuli (i.e., vertical bars) that simulated potential refuges from a dangerous situation. When presented pairs of stimuli differed in numerosity, snails showed a spontaneous preference for the larger group up to four versus five items. However, these results did not reach a firm conclusion on which information (i.e., discrete and continuous variables) were exploited by snails to make their decisions. Indeed, larger groups have more numerical units, as well as higher density, contour length, and cumulative surface area. It is not easy to disentangle the role of nonnumerical variables in quantity discrimination, especially when animals are tested for their spontaneous preferences (Henik 2016). However, Bisazza and Gatto (2021) conducted subsequent experiments by controlling the influences of continuous variables, such as the stimuli’s area, width, density, or orientation, thus it is implausible for *T. pisana* to use continuous variables as a proxy to estimate the number of stems present in a cluster. These evidences support the hypothesis of a numerical discrimination mechanism rather than that of a mechanism based on continuous quantity discrimination.

In addition, different continuous variables have been controlled in training procedures. Dacke and Srinivasan (2008) trained honeybees to forage in a tunnel. Bees can find a food reward after they have passed a specific number of landmarks when flying. Authors modified the distance of the food reward but kept the number of encountered landmarks constant. The findings showed that bees can memorize the number of landmarks encountered during flight before choosing the landmark in the previous trained position, even when the shape, size, or position of landmarks were changed, suggesting that they were capable of object-independent counting. Howard et al. (2018) trained honeybees (*Apis mellifera*) to make quantity discriminations based on the quantitative concepts of “greater than” or “less than”. They used white cardboard squares as stimuli with black elements differing for numerosity, configuration, shape, and size of elements for 82 different configurations. Individual honeybees learned the abstract rules and used those when solving novel tasks, demonstrating the first evidence of the “zero concept” in an invertebrate species.

Shaki and Fischer (2020) have recently criticized the experiments conducted by Howard et al. (2018, 2019a, b). They suggested that the appetitive–aversive learning procedure and lack of sufficiently demanding transfer tests might have led to biased reward structures and generated unclear results. In addition, Shaki and Fischer (2020) claimed that Howard et al. should have used identical outcome problems (e.g., 4 – 1 versus 2 + 1) during transfer testing to enable fair comparisons across arithmetic operations. Shaki and Fisher concluded that the available evidence does not yet conclusively demonstrate symbolic representations of numerosity, arithmetic abilities, or abstract concept acquisition in honeybees. Howard et al. (2020b) responded to the criticism by Shaki and Fischer (2020), claiming that they trained honeybees to discriminate visual displays with differing numbers of shapes by presenting thousands of different patterns and controlling them for continuous variables, such as convex hull, density, spatial arrangement, and cumulative surface area. MaBouDi et al. (2021) conducted an experiment in which they used the same methods used by Howard et al. (2018). The additional control experiments performed by MaBouDi et al. (2021) showed that bees could solve these tasks using non-numerical cues, such as spatial frequency. In addition, they developed a network model showing that, using biologically plausible visual feature filtering and a simple associative rule, bees were capable of learning the task using only continuous cues inherent in the training stimuli, without numerical processing at all. Their findings suggested that there was an alternative strategy that animals could have used in studies using two-dimensional displays of enumerated shapes and methods that did not control for all low-level cues. This puts into question the claims of the numerosity studies that did not control for spatial frequency or other low-level cues. These arguments are a further demonstration of the importance of controlling for nonnumerical variables before concluding that an animal has relied on numerical discrimination.

### Quantitative processing in small neural circuits

Complex cognitive function, such as quantitative ability, has been traditionally considered a prerogative to species with relatively large brains (Arsalidou and Taylor 2011). Therefore, many studies reported in this review show that quantitative abilities might be computed in a relatively small brain. Indeed, invertebrates show complex behavior, such as behavioral plasticity, spatial cognition, communication skills, and associative learning without a relative small number of neurons compared to the vertebrates (human: 85 billion, Azevedo et al. 2009; chimpanzee: 22 billion, Herculano-Houzel 2012; grey parrot: 1.57 billion, Olkowicz et al. 2016; mouse: 0.07 billion, Herculano-Houzel et al. 2006; zebrafish: 0.01 billion, Hinsch and Zupanc 2007; bees: 0.001 billion, Menzel and Giurfa 2001; snail: 0.00001 billion, Roth and Dicke 2005). Although they have differences in the number of neuron and neural architectures, invertebrates and vertebrates have developed similar solutions to process information. Thus, larger brains may be a consequence of processing information from larger sense organs, which enable function diversification of neurons but require a greater energy supply (Niven and Laughlin 2008; Chittka and Niven 2009).

Neuroimaging studies on primates and corvids have shown that the capacity of quantity discrimination did not require a dedicated cortical module (Nieder 2019), but this capacity may have been an inherent mechanism that relied on visual perception to generate a representation of the object of interest in the working memory. This mechanism required a neural circuit activated by threshold-sensory process and integrated-sensory inputs while observing the target object (Burr and Ross 2008; Dehaene and Changeux 1993; Stoianov and Zorzi 2012). Invertebrates do not have extended structured brain architectures as vertebrates do.

One necessary requirement for the neural circuit underlying numerical competence might be high-level brain areas that integrate sensory information. The general plan of the invertebrate nervous system consists of a ventral, ganglionated nerve cord and a dorsal-anterior ganglion, which usually lacks a pronounced organization and has no myelin sheath covering the nerve cells. However, increasing the diameter of the axon with local neural connections permits highly efficient conduction velocity, providing fast responses to external stimulation, such as the circuit underlying escape behavior (Burrows 1996). The segmented confirmation of ventral cord permits more movement freedom and faster responses to external stimuli in the solicited body region. Phyla including *Anellidae*, *Mollusca*, and *Arthropoda* present the most complex organization of the nervous system among invertebrates. Their dorsal–anterior ganglion consists of an organized single brain above the pharynx with defined front, middle, and back sections. Each section is usually designated to process and integrate different types of information. For example, the anterior section of arthropod brains—the protocerebrum—receives the innervation of visual organs; while the back section—the tritocerebrum—innervates the mouthparts and the initial trait of the digestive canal. However, a higher order area in bees’ brains might have the neural circuit at the base of numerosity representation. The central complex is a series of four interconnected neuropiles, which connected the midline of the protocerebrum (Chapman 1998). Its principal role is to process the visual information previously integrated from the optic lobes, which is involved during navigation and object recognition (Pfeiffer and Homberg 2014). Thus, it appears to be a valid candidate for processing quantitative information (Giurfa 2019). Further studies should address the involvement of brain structures in other invertebrate species (Giurfa 2019; Nieder 2021).

## Conclusions

Despite the increasing abundance of studies discussed in this review (Giurfa 2019; Skorupski et al. 2018; Nieder 2020), some issues remain largely unsolved before understanding quantitative ability in invertebrates. First, studies on invertebrates have mainly focused on investigating quantitative abilities to solve ‘more versus fewer’ comparison tasks in the small number range (see Howard et al. 2019a). Studies on vertebrates show different performance levels when individuals perform quantitative discrimination tasks between groups in the small number ranges (e.g., 1–4 items) and in the larger number ranges (e.g., items > 4; Feigenson et al. 2004). Several authors have suggested that vertebrates share two distinct systems for processing quantitative information. Recent studies on bees have suggested that honeybees may exhibit the numerical distance effect (Bortot et al. 2019; Howard et al. 2018, 2020a, b). For example, Bortot et al. (2019) explored the use of the absolute or relative numerosity rule to solve numerical discrimination tasks. Free-flying bees were trained to choose a stimulus (i.e., white cardboard) containing three dots by presenting one of two possible quantity discriminations (i.e., 2 vs. 3, or 3 vs 4) and then testing the novel one. The authors found a ratio-dependent effect because the bees’ accuracy was higher when discriminating stimuli containing two and three dots (numerical ratio: 0.67) rather than stimuli containing three and four dots (numerical ratio: 0.75). However, Gatto and Carlesso (2019) found no difference in discrimination performance when crickets were presented with a dichotomous choice between sets differing by numerosity in ratio function (i.e., 0.25, 0.50, and 0.67).

Although it has been suggested that nonsymbolic numerical systems have a long evolutionary history (Beran 2008; Feigenson et al. 2004), its origin is debated. The system might have originated in an ancient ancestor common to most distant phylogenetic species (homology) or different species that might evolve independently similar functional systems (homoplasy). Jones et al. (2014) attempted to solve this issue by comparing numerical performances of humans and primates (i.e., three lemur species: *Lemur catta*, *Eulemur mongoz*, and *Eulemur macaco flavifrons*; and one Old World monkey species: *Macaca mulatta*), using the same numerical task, range of numerical discrimination, and stimulus controls. Results revealed an equivalent performance among primates, reaching a qualitatively similar performance to that of humans. Neurobiological and behavioral studies reported similar findings to Jones and colleagues (see Agrillo et al. 2012; Hanus and Call 2007; Jordan and Brannon 2006), thus supporting the hypothesis of a homoplasy origin for numerical systems.

Numerical cognition was historically believed to be a unique prerogative of humans, but a plethora of studies showed that such capacity was widespread among vertebrates. Invertebrates demonstrated highly cognitive function similar to those of vertebrates, such as spatial cognition, sophisticated communication skill, and associative learning (Leadbeater and Chittka 2007; Chittka and Niven 2009). Thus, invertebrates could be a candidate model for investigating the evolutionary origin of the numerical system and the mechanism and neural circuit underlying such competence (Giurfa 2019; Nieder 2021). However, the knowledge of quantitative ability in invertebrates is mainly limited to arthropods and a few contexts (Table 1). Many more studies are necessary for understanding the mechanisms underlining the quantitative abilities in invertebrates.
